# Static and dynamic brain morphological changes in isolated REM sleep behavior disorder compared to normal aging

**DOI:** 10.3389/fnins.2024.1365307

**Published:** 2024-05-01

**Authors:** Gilsoon Park, Hyunjin Jo, Yaqiong Chai, Hea Ree Park, Hanul Lee, Eun Yeon Joo, Hosung Kim

**Affiliations:** ^1^USC Stevens Neuroimaging and Informatics Institute, Keck School of Medicine of USC, University of Southern California, Los Angeles, CA, United States; ^2^Department of Neurology, Neuroscience Center, Samsung Medical Center, Samsung Biomedical Research Institute, School of Medicine, Sungkyunkwan University, Seoul, Republic of Korea; ^3^Medical Research Institute, Sungkyunkwan University School of Medicine, Suwon, Republic of Korea; ^4^Department of Neurology, Inje University College of Medicine, Ilsan Paik Hospital, Goyang, Republic of Korea

**Keywords:** rapid eye movement sleep behavior disorder, longitudinal studies, cerebral cortical thinning, subcortical volume atrophy, MRI analysis

## Abstract

**Objective/background:**

To assess whether cerebral structural alterations in isolated rapid eye movement sleep behavior disorder (iRBD) are progressive and differ from those of normal aging and whether they are related to clinical symptoms.

**Patients/methods:**

In a longitudinal study of 18 patients with iRBD (age, 66.1 ± 5.7 years; 13 males; follow-up, 1.6 ± 0.6 years) and 24 age-matched healthy controls (age, 67.0 ± 4.9 years; 12 males; follow-up, 2.0 ± 0.9 years), all participants underwent multiple extensive clinical examinations, neuropsychological tests, and magnetic resonance imaging at baseline and follow-up. Surface-based cortical reconstruction and automated subcortical structural segmentation were performed on T1-weighted images. We used mixed-effects models to examine the differences between the groups and the differences in anatomical changes over time.

**Results:**

None of the patients with iRBD demonstrated phenoconversion during the follow-up. Patients with iRBD had thinner cortices in the frontal, occipital, and temporal regions, and more caudate atrophy, compared to that in controls. In similar regions, group-by-age interaction analysis revealed that patients with iRBD demonstrated significantly slower decreases in cortical thickness and caudate volume with aging than that observed in controls. Patients with iRBD had lower scores on the Korean version of the Mini-Mental Status Examination (*p* = 0.037) and frontal and executive functions (*p* = 0.049) at baseline than those in controls; however, no significant group-by-age interaction was identified.

**Conclusion:**

Patients with iRBD show brain atrophy in the regions that are overlapped with the areas that have been documented to be affected in early stages of Parkinson’s disease. Such atrophy in iRBD may not be progressive but may be slower than that in normal aging. Cognitive impairment in iRBD is not progressive.

## 1 Introduction

Isolated rapid eye movement (REM) sleep behavior disorder (iRBD) is a parasomnia characterized by the loss of physiological atonia of skeletal muscles and abnormal behavior during REM sleep (Dauvilliers et al., 2018). Previous studies have suggested that most patients with iRBD eventually develop a defined neurodegenerative disease, particularly α-synucleinopathies, such as Parkinson’s disease (PD), dementia with Lewy bodies, and multiple system atrophy (Wing et al., 2012; Schenck et al., 2013; Iranzo et al., 2014; Arnulf et al., 2015; Mahlknecht et al., 2015; Postuma et al., 2015; Li et al., 2017). According to a recent multicenter study, the overall conversion rate from iRBD to an overt neurodegenerative syndrome is 6.3% per year, with 73.5% of patients converting over a 12-year follow-up (Postuma et al., 2019). Additionally, a majority of individuals with iRBD are diagnosed with a synucleinopathy within 20 years of onset of iRBD (Postuma et al., 2019). This high phenoconversion rate and long prodromal time window of iRBD suggest that the iRBD population can serve as an ideal study group for neuroprotective trials against neurodegeneration (Postuma, 2022). Given the high phenoconversion rate in iRBD, several studies have aimed to identify biomarkers in them that can predict phenoconversion. However, since iRBD has a long prodromal window, it is important to understand whether and how iRBD progresses during this period. Particularly, considering that the typical age of onset of iRBD is >50 years, (Zhou et al., 2014) it is necessary to identify how iRBD progression is different from normal aging, which requires a longitudinal comparative study between patients with iRBD and healthy controls.

Structural magnetic resonance imaging (MRI) is a powerful tool for identifying the structural features of the brain in patients with iRBD. Longitudinal imaging studies allow us to view the temporal evolution of a disease and have the potential to assess drug efficacy (Sabuncu et al., 2011). Most MRI studies have attempted to assess gray matter (GM) abnormalities in patients with iRBD and agree that the primary structural changes in these patients occur in the deep GM nuclei, cortical GM, and cerebellum. For example, compared with controls, patients with iRBD demonstrate volume reduction in deep GMs, such as the putamen, (Ellmore et al., 2010; Rahayel et al., 2017) caudate nucleus, (Rahayel et al., 2017; Chen et al., 2020) external and internal left pallidum, (Rahayel et al., 2017) cerebellum, (Hanyu et al., 2012) cortical thinning in the lingual gyrus, (Rahayel et al., 2015) fusiform gyrus, (Rahayel et al., 2015; Campabadal et al., 2019) lateral occipital cortex, (Campabadal et al., 2019; Pereira et al., 2019) orbitofrontal cortex, (Rahayel et al., 2015, 2017) cingulate cortex, (Rahayel et al., 2015, 2017) dorsolateral frontal cortex, (Rahayel et al., 2015, 2017) and parietal cortex (Campabadal et al., 2019, 2020; Pereira et al., 2019). Furthermore, studies that compared iRBD and early PD with controls have reported that subtle structural changes, similar to the patterns seen in PD, were already present in patients with iRBD (Pereira et al., 2019). Furthermore, such MRI features appear to be significantly correlated with clinical and neuropsychological characteristics in patients with iRBD (Pereira et al., 2019). However, these cross-sectional structural studies did not assess whether such a relation persisted over time.

To the best of our knowledge, only three longitudinal MRI studies have been conducted in polysomnography (PSG)-confirmed iRBD. In a study that followed 27 patients with iRBD for approximately 3 years, the authors found that converters to Lewy body disorders had widespread thinning in the left superior frontal, right precentral, and right lateral occipital gyri compared to that in non-converters (Pereira et al., 2019). Another study followed 50 patients with iRBD for approximately 4.4 years and found that the mean whole-brain cortical thickness predicted phenoconversion in these patients with a hazard ratio of 9.33 (1.16–74.12) (Shin et al., 2023). However, these studies did not differentiate the effects of aging on cortical thinning from disease progression, mainly because they did not include follow-up data in healthy controls. Only one study has compared the progression of iRBD to normal aging in controls; the results of this study revealed that the iRBD group experienced more thinning in the superior parietal and precuneus, the cuneus, the occipital pole, and some areas in the lateral orbitofrontal and frontopolar cortices compared with that in controls (Campabadal et al., 2020). However, considering that the average age of onset of RBD is 61 years, (Olson et al., 2000) this study included patients with relatively late-onset iRBD (65.6 ± 7.5 years). Therefore, our study aimed to assess two main objectives by conducting a longitudinal study and comparing patients with iRBD having relatively early disease onset with healthy controls, in contrast to previous studies (58.2 ± 9.2 years vs. previous studies: 65.6 ± 7.5 years). The objectives were as follows: (1) determining whether patients with iRBD with relatively early disease onset demonstrate structural alterations of the brain, and (2) investigating whether these structural alterations are progressive. Additionally, we examined whether these changes were correlated with their neuropsychological performances. These efforts to clarify the progression of iRBD will help us comprehend its degenerative course.

## 2 Methods

### 2.1 Participants

Patient recruitment and data acquisition were performed at the Samsung Medical Center from March 2018 to October 2020. We excluded those with serious medical issues, concomitant neurological or psychiatric disorders, or difficulty in cooperating with the study process.

For the patient group, RBD was diagnosed using the third edition of the International Classification of Sleep Disorders criteria, (Sateia, 2014) and the Korean version of the RBD questionnaire (RBDQ-KR) was used to identify the status of RBD (You et al., 2017). Only patients with iRBD, without evidence of Parkinsonism or dementia, were included. Control participants were recruited from the outpatients of a general practitioner clinic. None of the patients had a history of sleep disorders, such as parasomnia or obstructive sleep apnea. Ultimately, 18 patients with iRBD and 24 controls were included in this study.

The interval between baseline and follow-up was 1.6 ± 0.6 years (mean ± standard deviation) in the patients and 2.0 ± 0.9 years in the controls (*p* = 0.97). All participants underwent extensive clinical examination, neuropsychological examination, and 3-Tesla MRI at each visit. We employed the Korean version of the Mini-Mental Status Examination and the Seoul Neuropsychological Screening Battery (SNSB) 2nd edition (Kang et al., 2003). PSG was performed only in patients with iRBD. The study was conducted in accordance with the guidelines of the Declaration of Helsinki and approved by the Institutional Review Board of Samsung Medical Center (IRB No. 2022–05-115).

### 2.2 Clinical evaluations

#### 2.2.1 Neurological evaluation

At every visit, the patients were examined for neurological disorders through detailed history-taking and neurological examinations by neurologists. A standardized motor examination was performed using the Korean version of the Unified Parkinson’s Disease Rating Scale (Park et al., 2020). Olfactory function was examined using the Korean version of the Sniffin’s Sticks test. Eight different smells were tested, with the patient choosing one smell from the four correct answers (0–4, anosmia; 5–6, hyposmia; and 7–8, normosmia) (Cho et al., 2009).

#### 2.2.2 Sleep study (PSG and questionnaires)

Patients with iRBD completed questionnaires on sleep-related measures [Epworth Sleepiness Scale (ESS), Insomnia Severity Index (ISI), and Pittsburgh Sleep Quality Index (PSQI)] and depressive symptoms [Korean-Beck Depression Inventory-II (K-BDI-II)] along with PSG. The method of recording and analysis of PSG and the interpretation of the above questionnaires were published previously (Jo et al., 2021). We calculated the proportion of epochs of REM sleep without atonia among the total REM sleep epochs and used it for analysis (total number of REM epochs with REM sleep without atonia/total REM sleep epochs × 100) based on the standard rule (Berry et al., 2012).

#### 2.2.3 Neuropsychological test

All subjects underwent the Korean version of the Mini-Mental Status Examination (Han et al., 2008) and Seoul Neuropsychological Screening Battery (SNSB) 2nd edition at baseline and follow-up (Kang et al., 2003). This battery is a comprehensive neuropsychological test that assesses five cognitive domains: attention (forward and backward Digit Span Test), language (Korean version of the Boston Naming Test), visuospatial function (Rey Complex Figure Test: copy), verbal and visual memory (Seoul Verbal Learning Test, SVLT: immediate and 20-min delayed recall and recognition, immediate and 20-min delayed recall and recognition), and frontal/executive functions (semantic and phonemic Controlled Oral Word Association Test); the Stroop test; the Digit Symbol Coding; the Korean version of the Trail-Making Test (Kang et al., 2003). Raw scores on the tests were transformed into *z*-scores by adjusting for age, sex, and years of education.

### 2.3 MRI acquisition and preprocessing

T1-weighted images of all participants were acquired using 3 T Philips Achieva scanner (repetition time = 9.8685 ms; echo time = 4.593 ms; slice thickness = 1 mm; flip angle = 8°; voxel size = 0.5 mm^3^ isotropic; FOV = 240 mm) at both times.

To estimate cortical thickness, we used the CIVET v2.1.0 pipeline, which includes sequential image processing steps to build a cortical surface model and extract the cortical thickness from the model^1^ (Collins et al., 1994; Sled et al., 1998; Zijdenbos et al., 1998; Macdonald et al., 2000; Smith, 2002; Tohka et al., 2004; Kim et al., 2005; Ad-Dab’bagh et al., 2006; Lyttelton et al., 2007). The final surface models were resampled to an MNI152 template space and smoothed using a Gaussian kernel with a 30-mm FWHM for group comparisons (Chung et al., 2003). We performed QC on the surface models of all participants using the verification image files generated by the Civet pipeline. None of the participants were excluded from the study.

We used deep neural networks, as reported by Park et al. (2021) for deep grey matter structure segmentation. This network structure with highlighting foreground (HF) modules was proposed to alleviate the commonly observed imbalanced data problems in medical image segmentation, where the foreground (target brain structure to segment) is smaller than the background (non-target brain and non-brain structures). This HF network demonstrated the best performance in the white matter hyperintensity segmentation challenge (team pgs, https://wmh.isi.uu.nl/#_Toc122355693). To adapt the model to the segmentation of deep gray matter, we chose the Parkinson’s Progression Markers Initiative, which included controls and RBD data as the training data. We randomly selected 42 participants from the dataset (21 controls and 21 patients with RBD). A neuroanatomist delineated the label information of the selected dataset. Using the external dataset, we performed 5-fold cross-validation to evaluate the segmentation performance. The HF network showed remarkable segmentation performance, which was comparable to the experts’ intra-rater reproducibility (mean dice score = 0.92). The mean dice score for the automated segmentation was 0.9211 (thalamus, 0.9477; caudate, 0.9417; putamen, 0.9435; pallidum, 0.9045; hippocampus, 0.9261; amygdala, 0.9069; and accumbens, 0.8772). Finally, we applied an ensemble of five models resulting from the 5-fold cross-validation to the current iRBD and control datasets. To correct the volume measurements for the total intracranial volume, we registered the participants with the MNI152 space and segmented the deep gray matter structures in that space.

### 2.4 Statistical analyses

Linear regression models were constructed to assess the brain structural alterations in the control and iRBD groups. Linear regression at each vertex or each deep GM structure was performed using SurfStat (Worsley et al., 2009) and MATLAB 2021b.^2^ First, we built a cross-sectional model to analyze the baseline brain structural volume (cortical thickness and deep GM volume) as a dependent variable and group information as an independent variable to compare baseline cortical thickness between the two groups.

We used mixed-effects models in our study to account for both fixed and random effects and to account for the longitudinal and nested data structure of our dataset. This approach allowed us to model individual variability while analyzing specific variables of interest, thereby increasing the validity and reliability of our findings. The mixed-effects models were used to analyze (1) group differences in cortical thickness and deep GM volumes; (2) group × age interaction to assess whether the effect of aging on cortical thickness and deep GM volumes differs between iRBD and control groups; (3) correlation of volumes with clinical parameters (SNSB and PSG); and (4) correlation of Δ volume (=follow-up-baseline/scan interval in months) with Δ clinical parameters. Sex, years of education, duration of iRBD, and age at scanning were included as covariates to control for confounding effects in all regression models. To adjust for multiple comparisons, we adopted a false discovery rate for all regression analyses.

## 3 Results

### 3.1 Demographics and clinical characteristics

Two-sample *t*-tests were performed to compare the demographic and clinical characteristics between the control and iRBD groups. At baseline, there was no difference between the iRBD and control groups in mean age (66.1 ± 5.7 vs. 67.4 ± 5.6 years, respectively; *p* = 0.482) and education level (13.1 ± 4.6 vs. 13.0 ± 3.8 years, respectively; *p* = 0.932). The proportion of men was higher in the iRBD group than in the control group (72.2% vs. 50.0%, respectively; Fisher’s exact test, *p* < 0.001). In patients with iRBD, the mean age at RBD onset was 58.2 ± 9.2 years and the mean disease duration was 6.7 ± 5.8 years. Out of 18 patients with iRBD, only one was on therapy for RBD at baseline (clonazepam, 0.25 mg). Of the 17 patients who were not on therapy initially, two had declined treatment, and the remaining 15 patients started therapy after the baseline test. During the follow-up period, 12 patients were on therapy with an average of 1.2 mg of melatonin and 0.4 mg of clonazepam.

The interval between the baseline and follow-up was 1.6 ± 0.6 years (mean ± standard deviation) in the patients and 2.0 ± 0.9 years in controls, which was not significantly different (*p* = 0.97). There was no difference in the mean age between the iRBD and control groups at the time of follow-up (67.7 ± 6.0 vs. 69.3 ± 5.8 years, respectively; *p* = 0.393). None of the patients with iRBD showed phenoconversion during the follow-up period. Detailed information is provided in Table 1.

**Table 1:** Demographics and clinical characteristics of the participants.

	iRBD (*n* = 18)	HC (*n* = 24)	*p*-value
Age at baseline, years	66.1 ± 5.7	67.4 ± 5.6	0.4817
Follow-up duration, years	1.64 ± 0.63	1.99 ± 0.95	0.1844
Age at follow-up, years	67.7 ± 6.0	69.3 ± 5.8	0.3931
Sex (M/F) (%)	13/18 (72.2)	12/24 (50.0)	0.1450
Education, years	13.1 ± 4.6	13.0 ± 3.8	0.9322
Age at RBD onset, years	58.2 ± 9.2		
RBD duration since symptom onset, years	6.7 ± 5.8		
RBDQ_KR	46.1 ± 22.9		
KVSS	4.7 ± 0.9		

### 3.2 Volumetric analysis on MRI

#### 3.2.1 Cortical thickness

##### 3.2.1.1 Group differences between iRBD and control groups

**Figure 1:**
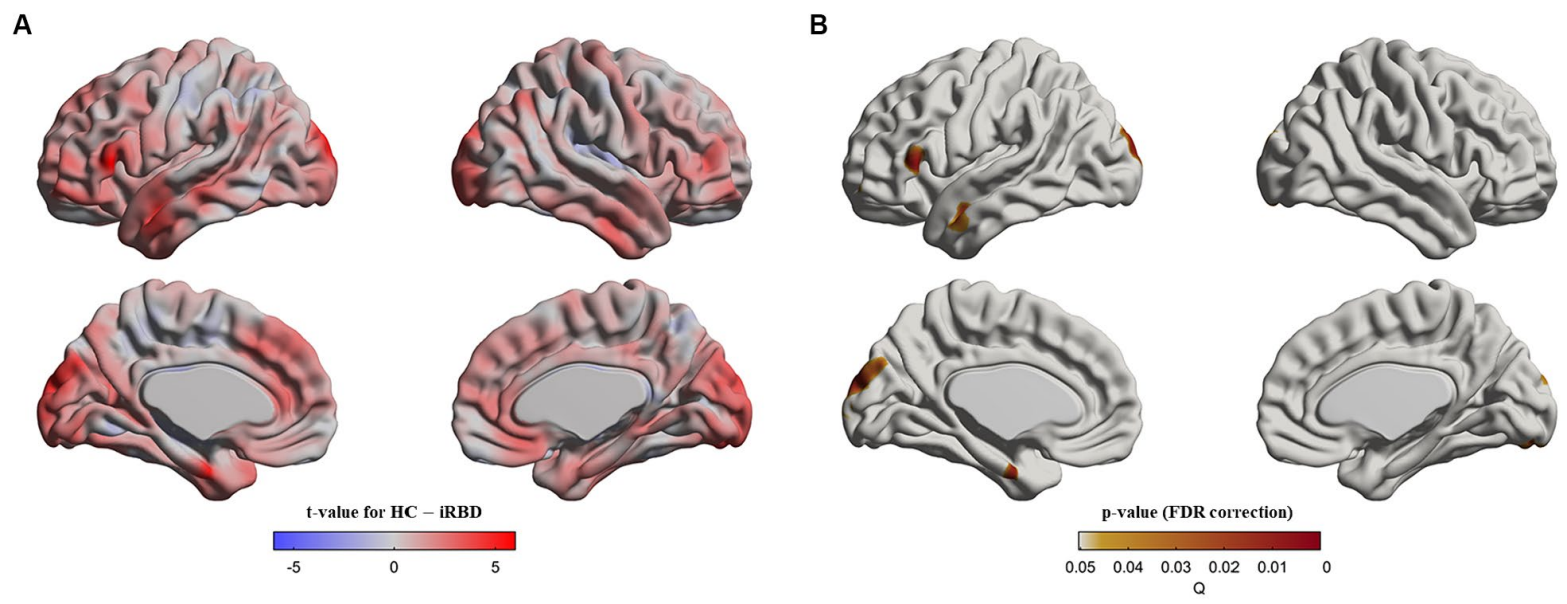
Cortical thinning in idiopathic REM sleep behavior disorder (iRBD) patients compared to healthy control (HC) group at baseline. Vertex-wise group comparisons of cortical thickness between iRBD and HC groups were performed. To remove confounding effects, we used age, sex, education years, and iRBD duration as covariance. **(A)** *T*-value map representing the tested contrast of the HC group minus the iRBD group. **(B)** *P*-value map after false discovery ratio (FDR) correction. Specifically, iRBD patients displayed cortical thinning in the left inferior frontal gyrus, left superior/middle temporal gyri, left superior occipital gyrus, and bilateral cuneus compared to HC.

At baseline, patients with iRBD demonstrated cortical thinning in the left inferior frontal gyrus, left superior/middle temporal gyri, left superior occipital gyrus, and bilateral cuneus compared with the controls (*p* < 0.05, after false discovery rate correction, Figure 1).

When the baseline and follow-up scans were pooled and analyzed using a mixed effect model, we observed that patients with iRBD had thinner cortical thickness than those in the controls, in the same regions as seen at baseline as well as additional cortical thinning in the temporal (left middle and inferior temporal gyrus, left temporal pole, and right middle and inferior temporal gyrus), frontal (left medial superior frontal gyrus, right middle frontal gyrus, right anterior cingulate gyrus, and paracingulate gyri), and occipital (left calcarine fissure and surrounding cortex and right cuneus) areas (Supplementary Figure S1).

##### 3.2.1.2 Group-by-age interaction

**Figure 2:**
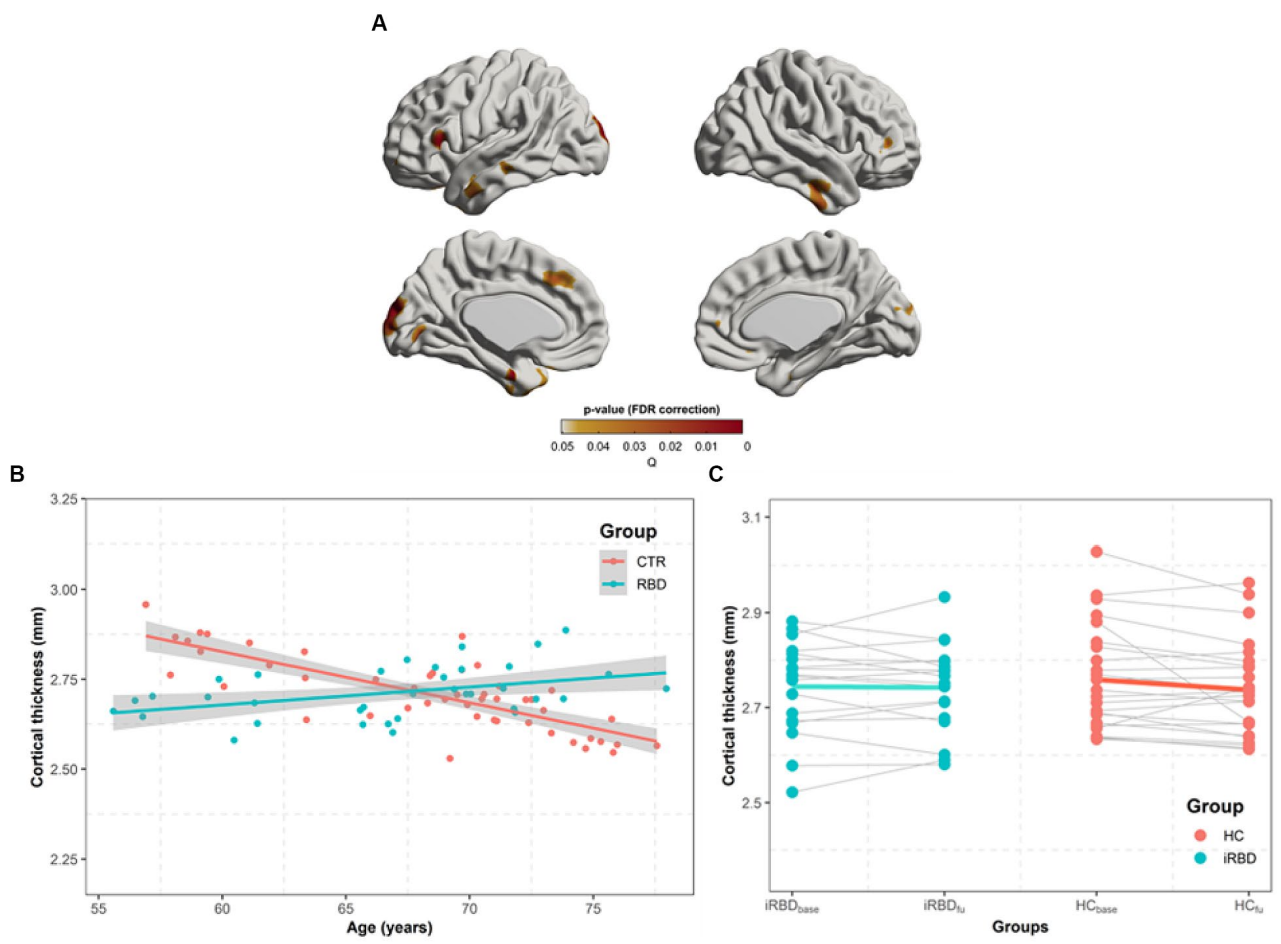
Progression of cortical thickness changes in idiopathic REM sleep behavior disorder (iRBD) patients and healthy control (HC) group. Longitudinal patterns of cortical thickness were investigated in iRBD and HC groups by analyzing the group-by-age interaction term using mixed-effects models built on baseline and follow-up measurements. Subject effects due to multiple measurements were removed as a random effect in these models. To eliminate other confounding effects, sex, education years, iRBD duration, and age were included as covariates. **(A)** *p*-value map obtained by testing group-by-age interaction effects after false discovery ratio (FDR) correction. **(B)** In the significant region found in **(A)**, we averaged cortical thickness measurements per each subject at baseline and follow-up. The averaged values are plotted along with aging. Turquoise and red dots indicate individual cortical thickness values in iRBD and HC groups, respectively. Turquoise and red lines represent the linear fit of turquoise and red dots, respectively. The gray region represents the 95% confidence interval. **(C)** Each dot represents each subject’s averaged cortical thickness. Turquoise and red lines represent annual cortical thickness changes for each iRBD and HC group, respectively. Thin gray lines represent each pair of data. In **(B,C)**, the analysis of *group x age* revealed that the HC group had faster decreases in cortical thickness along with aging than iRBD patients did.

In similar regions observed in the group difference (Figure 2A), our analysis of the group x age interaction revealed that the control group demonstrated a faster decline in cortical thickness with age than that in patients with iRBD (Figures 2B,C).

#### 3.2.2 Subcortical volume analysis

In the subcortical volume analysis, significant caudate atrophy was found in the iRBD group compared to that in the control group (*p* = 0.04). A significant group-by-age interaction was observed in the caudate volume with a slower volume decrease in the iRBD group than that in the control group (Table 2). Similar to the results of the cortical thickness analysis, the initial atrophy of the caudate due to iRBD was significantly greater than that in controls; however, thereafter, it demonstrated a slower decrease compared to the decrease during normal aging (Supplementary Figure S2).

**Table 2:** Results of the group comparison of subcortical volumes and the analysis of the group-by-age interaction term.

	Volume ± std (cm^3^)		Group × Age interaction	
	HC	iRBD	*p*-value (corrected)	Beta
Thalamus	16.086 ± 1.499	15.951 ± 1.051	0.2916	0.0720
Caudate	8.933 ± 0.922^*^	8.853 ± 0.928	0.0392^*^	0.0958
Putamen	11.079 ± 1.196	11.225 ± 0.921	0.1211	0.0830
Pallidum	4.291 ± 0.573	4.564 ± 0.338	0.2916	0.0289
Hippocampus	9.611 ± 1.011	9.421 ± 1.022	0.7819	−0.0242
Amygdala	3.597 ± 0.271	3.697 ± 0.280	0.1211	0.0229
Accumbens	0.931 ± 0.154	0.884 ± 0.151	0.7212	0.0043

Mixed-effects models were adopted for these analyses. Subject effects due to multiple measurements were removed as a random effect in these models. To eliminate other confounding effects, sex, education years, iRBD duration, and age were included as covariates. ^*^p-value corrected by FDR < 0.05.

The observed cortical thinning affected areas involved in memory, vision, and higher-order functions like planning and judgment, along with the caudate which is important for motor control and learning.

### 3.3 Association with clinical profile

#### 3.3.1 Comparison of cognitive function between iRBD and controls

At baseline assessment, patients with iRBD demonstrated a lower KMMSE score than that in the controls (*p* = 0.034). However, there were no differences between the two groups in the items of the SNSB. At follow-up, patients with iRBD demonstrated poorer performance than those in the controls, in the frontal and executive functions (composite score, *p* = 0.002). Specifically, “Controlled Oral Word Association Test, phonemic” scores were significantly lower (*p* = 0.004). Additionally, iRBD patients exhibited cognitive decline compared to healthy controls. The KMMSE scores of patients with iRBD were lower than those of the controls (*p* = 0.04) (Supplementary Table S1). In the group analysis using a mixed-effects model, no significant results were found for group-by-age interactions.

#### 3.3.2 Association between cognitive function and cortical thickness and subcortical volume

No significant association was found between each cognitive function item/domain and cortical thickness in the region where a statistically significant difference in cortical thickness was found between the control and iRBD groups. In the subcortical volumes, a negative correlation was found between the accumbens volume and SVLT immediate recall score (*b* = −0.0304, *p* = 0.014) in the pooled group of iRBD and controls.

#### 3.3.3 Changes in sleep-related parameters and association with cortical thickness in patients with iRBD

In patients with iRBD, although the ISI score was lower at follow-up than that at baseline, other sleep-related indicators did not differ in this interval (Supplementary Table S2). There were no associations between the morphological measurements (cortical thickness and subcortical volume) and PSG parameters in patients with iRBD.

## 4 Discussion

Our study revealed that brain morphology in patients with iRBD differs from that in healthy controls and that it progresses differently from normal aging during the prodromal phase of iRBD. Specifically, patients with iRBD demonstrated a significantly thinner cortex and lower caudate volume than that observed in controls. However, this initial brain atrophy related to iRBD may be impeded during aging compared to that during normal aging. In neuropsychological tests, initial poor cognitive function was observed in patients with iRBD compared to that in the controls; however, there was no significant decline with aging. Additionally, a significant association between cognitive function scores and subcortical volume was noted.

### 4.1 Cortical thickness

In group comparisons, patients with iRBD demonstrated cortical thinning in the occipital, temporal, and frontal areas compared with that in the controls. Most previous studies on cortical thickness have found evidence of both anterior and posterior cortical degeneration in patients with iRBD. Campabadal et al. (2019) and Pereira et al. (2019) reported a parieto-occipital thinning pattern, and this cortical thinning pattern was similar to that observed in patients with newly diagnosed PD. Furthermore, Rahayel et al. (2015, 2017) reported cortical thinning in the frontal and parietal regions, and longer RBD duration and younger age of onset were related to cortical thinning in these regions. In our analysis of baseline MRI, patients with iRBD demonstrated cortical thinning in the occipital area, which is consistent with previous studies; additionally, cortical thinning in the temporal area was observed. This temporo-occipital atrophy pattern overlaps with RBD-related covariance pattern characterized as metabolic decreases in the occipital and temporal cortices on 18-fluorodeoxyglucose positron emission tomography (Wu et al., 2014). Interestingly, similar to our results, inferior temporal cortical thinning was observed in *de novo* PD studies (Pereira et al., 2014, 2019). Additionally, such thinning was reported in patients with PD and RBD compared with that in patients with PD without RBD (Rahayel et al., 2019; Yoon and Monchi, 2021). Our finding of thinning of the inferior temporal cortex is in line with previous studies and supports the importance of the temporal lobe in RBD, *de novo* PD, and PD with RBD. Although cortical thinning in the frontal area was observed in our study, it was not as extensive as that observed by Rahayel et al. (2019), presumably due to the shorter RBD duration. Our results demonstrated more extensive cortical thinning in the left hemisphere. Such asymmetric cortical thinning patterns have been reported in RBD, *de novo* PD, and PD with RBD (Pereira et al., 2019; Yoon and Monchi, 2021). The cortical thinning pattern observed in our study was similar to the findings of previous studies on *de novo* PD and patients with PD and RBD, thus suggesting that subtle PD-like GM changes may already be present in patients with iRBD.

In the group-by-age interaction analysis, patients with iRBD demonstrated significantly slower decreases in cortical thickness with aging compared to that in healthy controls in similar regions. To the best of our knowledge, Campabadal et al.’s (2020) study is the only longitudinal analysis to report GM loss in patients with iRBD compared to that in healthy controls. They reported that compared to that demonstrated by the controls, patients with iRBD had significantly greater progressive cortical thinning in the bilateral superior parietal and precuneus, right cuneus, left occipital pole, and lateral orbitofrontal gyri (Campabadal et al., 2020). Several reasons can be attributed to the conflicting results between their study and the current study. First, the previous study included patients with a relatively late onset of RBD than in our study (65.6 ± 7.5 years vs. 58.2 ± 9.2 years, respectively). Additionally, the authors found that cortical loss in posterior regions after a mean follow-up of 1.6 years was associated with late onset iRBD. In other words, their findings suggested that the later the age of RBD onset, the faster the disease progression. Our findings suggest that patients with early onset iRBD may initially experience greater neurodegeneration and then experience slower progression. A similar pattern has been observed in patients with AD, in which AD-specific brain atrophy follows a sigmoidal pattern with an initial accelerated phase followed by a decelerated phase during the later stages of the disease (Sabuncu et al., 2011). Specifically, the rate of cortical thinning accelerated throughout the presymptomatic and early MCI stages, starting from a level indistinguishable from cognitively normal participants and reaching the fastest pace at a stage with an MMSE score of approximately 21. However, AD-specific cortical thinning began to slow down subsequently. One explanation for such an early accelerated phase is the cumulative diffusion model, (Bass, 2004) which predicts that the rate of atrophy is proportional to that of aggregated atrophy (i.e., tissue loss at a location is aggravated by accumulating damage in its neighborhood). According to the cumulative diffusion model, atrophy accelerates initially, and the rate peaks at the point at which half of the potential tissue loss occurs. AD-specific brain atrophy is characterized by early acceleration, possibly driven by cumulative insults, such as amyloid toxicity, tangle deposition, and neuronal and synaptic dysfunction, followed by late deceleration, which is constrained by diminishing residual intact tissue (Sabuncu et al., 2011).

### 4.2 Subcortical volume

Over the last decade, volumetric and VBM studies have consistently identified a reduced volume in the caudate nucleus in patients with iRBD relative to that in controls (Rahayel et al., 2017; Chen et al., 2020). Consistent with previous studies, baseline MRI in our study revealed significant caudate atrophy in patients with iRBD compared to that in controls. However, these studies did not consider the age-related cell loss that occurs over time in subcortical structures. Only one longitudinal study analyzed subcortical GM volume; however, no differences were noted over time between the iRBD and control groups (Campabadal et al., 2020). However, our group-by-age interaction results revealed a slower volume decrease of the caudate in patients with iRBD than that in controls. Similar to the results of the cortical thickness analysis, the initial atrophy of the caudate due to iRBD was significantly greater than that in healthy controls; however, the caudate atrophy subsequently demonstrated a slower decrease compared with normal aging. This observation can be explained by the same context as that in cortical thinning patterns.

### 4.3 Neuropsychological test

In the current study, we found significant differences in the KMMSE scores and frontal executive function at baseline between patients with iRBD and healthy controls; however, no significant intergroup differences were observed.

Although previous longitudinal studies that included follow-up data in patients with iRBD reported a significant worsening of visuospatial learning, (Fantini et al., 2011) non-verbal logic, (Terzaghi et al., 2013) attention, (Terzaghi et al., 2013) executive functions, (Terzaghi et al., 2013) and working memory, (Youn et al., 2016) these studies did not consider the effects of aging. Only one study considered the effects of aging, and the authors found a cognitive decline in visual-form discrimination in patients with iRBD compared to that in healthy controls (Campabadal et al., 2020).

In our study, a significant difference was found between the iRBD and control groups but no significant group-by-age interaction could help explain the structural MRI results. We believe that the initial larger structural degeneration due to iRBD affects cognitive function and causes initial cognitive degeneration, which resulted in inter-group differences. Subsequently, the cognitive decline was similar between the iRBD and healthy control groups; therefore, no group-by-age difference was found.

### 4.4 Association between clinical profile and MRI measures

In regions with significant cortical thinning in the iRBD group, no significant associations with cognitive performance were found. This could be due to the small sample size or a lack of a linear relation between structural changes and neuropsychological impairment. However, in association studies of deep GM volumes and clinical profiles, we observed a relation between the volume of the nucleus accumbens (NA) and the SVLT immediate recall score (*b* = −0.0304, *p* = 0.014).

A growing body of literature suggests that NA is involved in learning and memory. Studies have found that the NA may be involved in declarative or hippocampal formation-dependent learning because the hippocampal formation projects extensively to the NA. Furthermore, as part of a larger striatal system, NA may be involved in learning and consolidation, but not in retrieval (Setlow, 1997). Damage to the NA and surrounding brain structures causes anterograde amnesia, a condition in which the ability to acquire new declarative memories is impaired while memories acquired prior to the damage are relatively spared (Phillips et al., 1987; Deluca and Diamond, 1995). Our finding that the volume of NA was only related to the immediate recall of verbal memory supports those of previous reports that NA plays a selective role in learning.

## 5 Limitations

Our study had several limitations. First, the small sample size stemmed from the difficulty in recruiting patients due to a longitudinal prospective study design, which requires a larger case study for confirmation. Second, the iRBD group had a higher proportion of males than that in the controls because RBD has a male predominance. However, this issue was mitigated by including sex as a covariate in the linear regression analysis. Third, we did not perform polysomnographic evaluation in the control group. Therefore, RBD or other sleep disorders, such as obstructive sleep apnea, could be underestimated. Fourth, none of the participants underwent phenoconversion during the short follow-up period. Long-term follow-up is required to confirm these findings. However, it is meaningful to confirm that, even during this short follow-up period, iRBD resulted in structural and cognitive changes that are distinct from those of normal aging. Finally, although there are no reports on any direct effects of melatonin and clonazepam on RBD progression, the potential role of treatment in patients with iRBD may have influenced the results.

## 6 Conclusion

Compared with healthy controls, patients with iRBD have brain atrophy in regions that overlap with regions reported previously in early PD. However, when compared to normal aging process, significant atrophy and cognitive decline were initially observed at baseline in iRBD but did not progress with aging as much as in normal aging subjects. Given our results, we surmised that structural changes in iRBD with relatively early disease onset may worsen significantly in the early stages of the disease but do not deteriorate any faster thereafter compared to normal aging, which is contrary to previous findings in patients with late onset of iRBD. This highlights the necessity for future large-sample research into the progression pattern of iRBD with aging. Nevertheless, our findings support the presence of multiple subtypes of iRBD, each of which potentially exhibits distinct progression trajectories toward neurodegenerative diseases such as PD.

## Data availability statement

The original contributions presented in the study are included in the article/Supplementary material, further inquiries can be directed to the corresponding authors.

## Ethics statement

The studies involving humans were approved by Institutional Review Board of Samsung Medical Center (IRB No. 2022–05-115). The studies were conducted in accordance with the local legislation and institutional requirements. The participants provided their written informed consent to participate in this study.

## Author contributions

GP: Data curation, Formal analysis, Methodology, Software, Writing – original draft. HJ: Formal analysis, Funding acquisition, Writing – original draft. YC: Methodology, Validation, Writing – review & editing. HP: Investigation, Supervision, Writing – review & editing. HL: Supervision, Validation, Writing – review & editing. EJ: Conceptualization, Funding acquisition, Resources, Supervision, Writing – review & editing. HK: Funding acquisition, Methodology, Supervision, Writing – review & editing.

## Footnotes

^1^ http://www.bic.mni.mcgill.ca/ServicesSoftware/CIVET

^2^ https://www.mathworks.com/
