# Supplementary material for: Static and dynamic brain morphological changes in isolated REM sleep behavior disorder compared to normal aging
Source: Front Neurosci. 2024 May 1;18:1365307. doi: 10.3389/fnins.2024.1365307 (PMC11094219; doi:10.3389/fnins.2024.1365307)
Supplement: Supplementary file 1 [file Data_Sheet_1.docx]

**Supplemental figures**


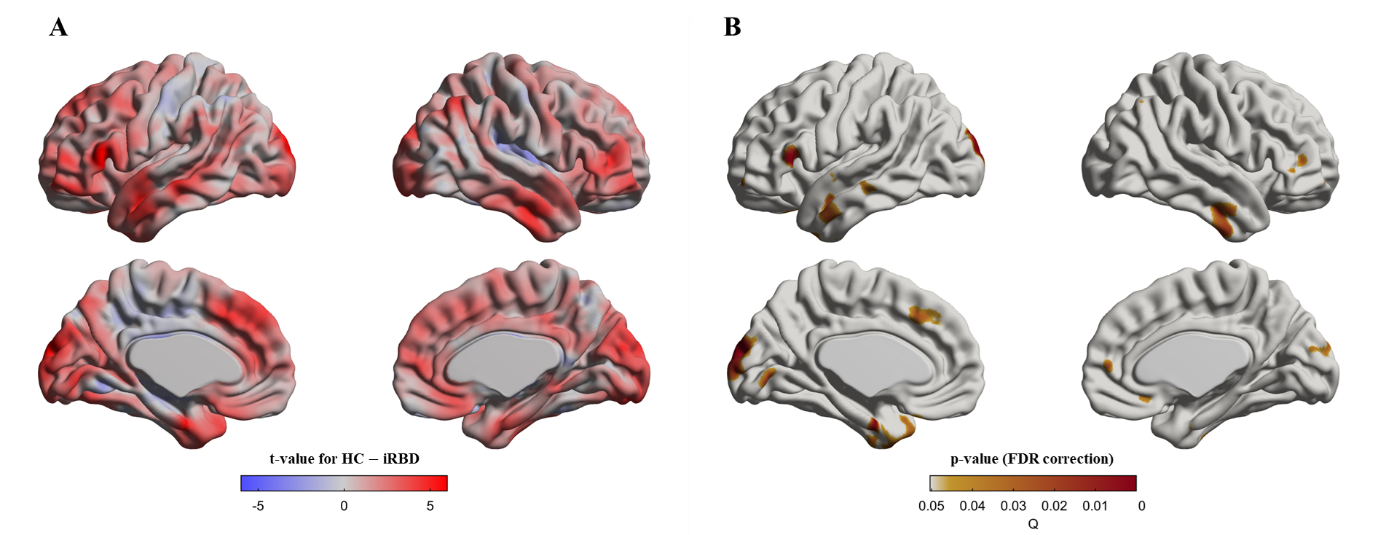


**Figure S1.** Cortical thinning in idiopathic REM sleep behavior disorder (iRBD) patients compared to healthy control (HC) group. Mixed-effects models were adopted to analyze baseline and follow-up measurements of each subject. Vertex-wise group comparisons of cortical thickness between iRBD and HC groups were performed. Subject effects due to multiple measurements were removed as a random effect in these models. To eliminate other confounding effects, sex, education years, iRBD duration, and age were included as covariates. **A.** t-value map representing the tested contrast of the HC group minus the iRBD group. **B.** p-value map after false discovery ratio (FDR) correction. We observed that iRBD patients had thinner cortical thickness than HC in the same regions as seen at baseline in Figure 1 as well as additional cortical thinning in temporal (left middle and inferior temporal gyrus, and left temporal pole, and right middle and inferior temporal gyrus), frontal (left medial superior frontal gyrus, right middle frontal gyrus, right anterior cingulate gyrus and paracingulate gyri), and occipital (left calcarine fissure and surrounding cortex and right cuneus) areas


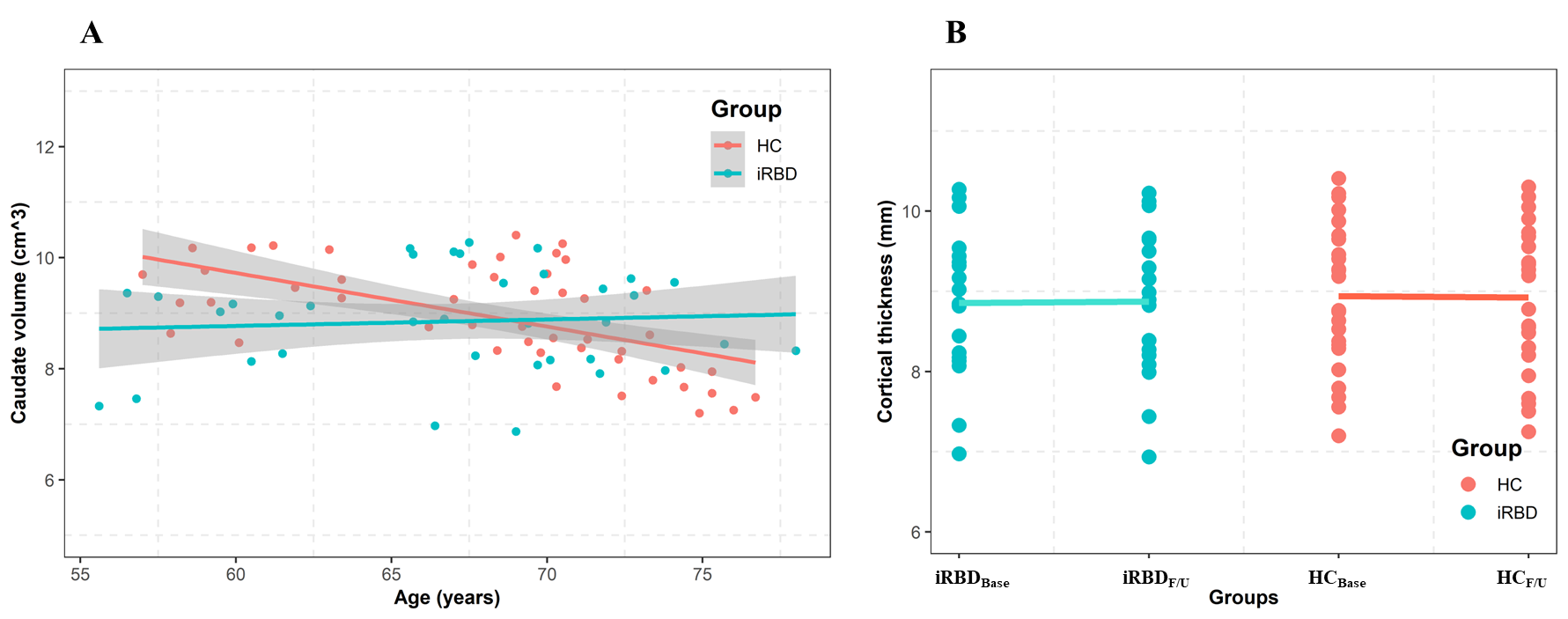


**Figure S2.** Progression of caudate volume changes in idiopathic REM sleep behavior disorder (iRBD) patients and healthy control (HC) group. Longitudinal patterns of caudate volume were investigated in iRBD and HC groups by analyzing the group-by-age interaction term using mixed-effects models built on baseline and follow-up measurements. Subject effects due to multiple measurements were removed as a random effect in these models. To eliminate other confounding effects, sex, education years, iRBD duration, and age were included as covariates. **A.** Caudate volumes are plotted along with aging. Turquoise and red dots indicate individual caudate volumes in iRBD and HC groups, respectively. Turquoise and red lines represent the linear fit of turquoise and red dots, respectively. The gray region represents the 95% confidence interval. **B.** Each dot represents each subject's caudate volume. Turquoise and red lines represent annual caudate volume changes for each iRBD and HC group, respectively. In **A-B**, the analysis of *group x age* revealed that the HC group had faster decreases in caudate volume along with aging than iRBD patients did.

**Supplemental tables**

**Table S1.** Comparison of the Seoul Neuropsychological Screening Battery (SNSB) between iRBD and HC groups

| **SNSB (z-scores)** | **Baseline** | | | **Follow-up** | | |
| --- | --- | --- | --- | --- | --- | --- |
|  | **iRBD** | **HC** | **p-value** | **iRBD** | **HC** | **p-value** |
| **Attention^†^** | 0.359±0.953 | 0.245±1.148 | 0.737 | 0.498±1.093 | 0.164±0.999 | 0.309 |
| Digit span forward | 0.383±1.021 | 0.316±1.110 | 0.842 | 0.311±1.037 | 0.305±1.076 | 0.986 |
| Digit span backward | 0.334±1.134 | 0.174±1.581 | 0.72 | 0.685±1.302 | 0.023±1.230 | 0.1 |
|  |  |  |  |  |  |  |
| **Language** |  |  |  |  |  |  |
| Korean-Boston naming test | 0.054±0.836 | 0.316±0.504 | 0.222 | 0.496±0.641 | 0.645±0.501 | 0.411 |
|  |  |  |  |  |  |  |
| **Visuospatial** |  |  |  |  |  |  |
| Rey complex figure test (copy) | -0.043±0.860 | 0.013±0.754 | 0.827 | 0.116±0.641 | 0.315±0.612 | 0.314 |
|  |  |  |  |  |  |  |
| **Verbal memory^†^** | 0.050±0.655 | 0.288±0.648 | 0.254 | 0.213±0.646 | 0.355±0.796 | 0.55 |
| Seoul verbal learning test  (immediate) | 0.227±0.826 | 0.399±1.029 | 0.566 | 0.161±0.665 | 0.346±1.114 | 0.544 |
| Seoul verbal learning test  (delayed responses) | -0.178±1.076 | 0.137±0.800 | 0.288 | 0.192±0.965 | 0.415±0.892 | 0.449 |
| Seoul verbal learning test  (recognition) | 0.102±0.774 | 0.327±0.810 | 0.374 | 0.287±0.700 | 0.150±0.987 | 0.628 |
|  |  |  |  |  |  |  |
| **Visual memory^†^** | 0.307±0.578 | 0.268±0.502 | 0.822 | 0.274±0.605 | 0.517±0.790 | 0.289 |
| Rey complex figure test  (immediate) | 0.483±0.682 | 0.353±0.870 | 0.605 | 0.359±0.832 | 0.583±0.875 | 0.406 |
| Rey complex figure test  (delayed) | 0.342±0.746 | 0.176±0.842 | 0.515 | 0.367±0.826 | 0.508±0.808 | 0.582 |
| Rey complex figure test  (recognition) | 0.095±0.797 | 0.276±0.737 | 0.456 | 0.097±0.577 | 0.572±1.314 | 0.161 |
|  |  |  |  |  |  |  |
| **Frontal and executive function^†^** | 0.111±0.611 | 0.272±0.594 | 0.467 | 0.068±0.426 | 0.545±0.458 | **0.002*** |
| Digit symbol test | 1.029±0.507 | 1.006±0.936 | 0.931 | 0.856±0.831 | 1.087±0.879 | 0.424 |
| Controlled oral word association test  (phonemic) | -0.120±0.949 | 0.195±1.436 | 0.445 | -0.490±0.722 | 0.534±1.257 | **0.004*** |
| Controlled oral word association test  (semantic, animal) | 0.238±1.281 | 0.182±0.967 | 0.873 | -0.005±1.046 | 0.475±1.175 | 0.177 |
| Controlled oral word association test  (semantic, supermarket) | 0.214±1.148 | 0.214±0.937 | 1 | -0.061±0.933 | 0.135±0.860 | 0.484 |
| Trail making test A,  time to completion | 0.314±0.786 | 0.687±0.460 | 0.126 | 0.432±0.517 | 0.680±0.392 | 0.102 |
| Trail making test B,  time to completion | -0.204±1.012 | 0.440±0.757 | 0.056 | 0.218±0.595 | 0.484±0.526 | 0.152 |
| Stroop test color, correct responses | 0.225±0.700 | 0.279±0.798 | 0.823 | 0.124±0.947 | 0.602±0.762 | 0.078 |
|  |  |  |  |  |  |  |
| **K-MMSE** | -0.208±1.085 | 0.378±0.600 | **0.034*** | -0.185±0.764 | 0.319±0.733 | **0.04*** |

*****: uncorrected p-value < 0.05, **^†^**: composited z-score

**Table S2**. Comparison of sleep-related parameters in iRBD patients

| **PSG** | **Baseline** | **Follow-up** | **p-value** |
| --- | --- | --- | --- |
| ESS | 5.77±3.17 | 5.23±3.19 | 0.347 |
| ISI | 9.60±7.07 | 6.40±6.74 | **0.023*** |
| PSQI | 6.64±4.84 | 5.45±3.08 | 0.168 |
| K-BDI-II | 12.25±6.68 | 9.92±5.95 | 0.189 |
|  |  |  |  |
| Total sleep time, min | 365.32±80.00 | 340.53±58.34 | 0.298 |
| Sleep latency, min | 10.72±11.24 | 21.23±22.32 | 0.114 |
| REM sleep latency, min | 82.25±54.51 | 107.36±54.40 | 0.142 |
| Sleep efficiency, % | 80.42±15.76 | 77.71±12.70 | 0.547 |
| N1 sleep time, % | 21.15±13.92 | 22.75±13.57 | 0.703 |
| N2 sleep time, % | 56.36±11.05 | 57.39±12.08 | 0.786 |
| N3 sleep time, % | 2.16±5.43 | 4.30±6.45 | 0.151 |
| REM sleep time, % | 20.57±10.07 | 15.83±5.05 | 0.103 |
| WASO, % | 18.15±16.00 | 18.34±12.46 | 0.969 |
| Apnea-hypopnea index, /h | 10.54±10.46 | 9.45±6.14 | 0.666 |
| Arousal index, /h | 20.40±9.63 | 18.54±8.06 | 0.500 |
| PLM index, /h | 37.14±22.14 | 47.09±33.32 | 0.304 |

*****: uncorrected p-value < 0.05
